# Supplementary material for: Development, Analysis, and Sensory Evaluation of Improved Bread Fortified with a Plant-Based Fermented Food Product
Source: Foods. 2023 Jul 25;12(15):2817. doi: 10.3390/foods12152817 (PMC10417715; doi:10.3390/foods12152817)
Supplement: Supplementary file 1 [file foods-12-02817-s001.zip › foods-2478730-supplementary.pdf]

Supplementary Material

Table S1. Nutritional and physicochemical profile of FFP and bread flour

	FFP	Bread flour (Gold Medal)**
Calories (kcal/100g of product)	467.6	366
Total Carbohydrates (%)	53.1	73.3
Dietary fiber (%)	42.6*	< 3
Proteins (%)	44.5	10
Fats (%)	2.4	0
Moisture Content (%)	12.8	NA
pH	4.4	NA
Mean Particle Size (mm)	0.1	NA

Information provided by the manufacturer. Protein content was determined by a combustion method according to AACC Method 46-30 (% N×6.25); *Dietary Fibre was determined as Total Dietary Fiber (TDF using the Megazyme enzymatic procedure (AACC approved method 441.93); moisture content was evaluated using AACC Method 44-15. **This information was obtained from the manufacturer's website (https://www.goldmedalflour.com/our-flour/bread-flour/). FFP: fermented food product; NA: not available.

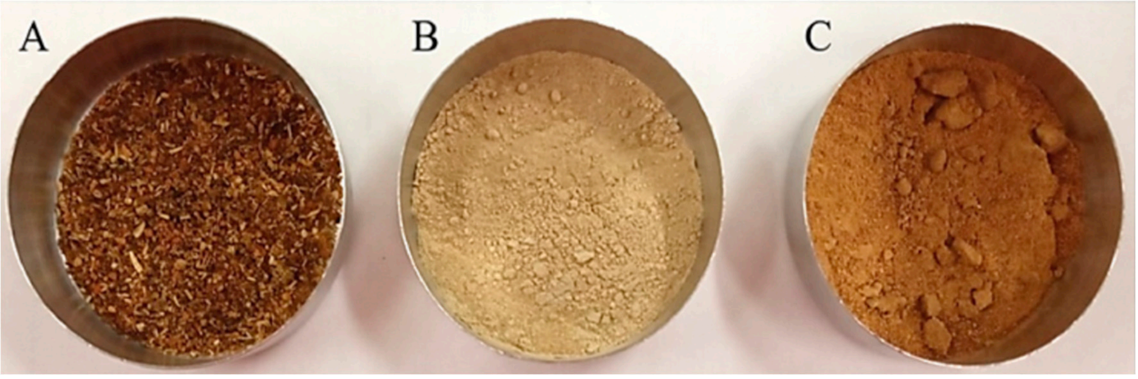

Supplementary Figure S1. Images of (A) unprocessed, (B) milled, (C) milled and autoclaved FFP. FFP: Fermented food product.

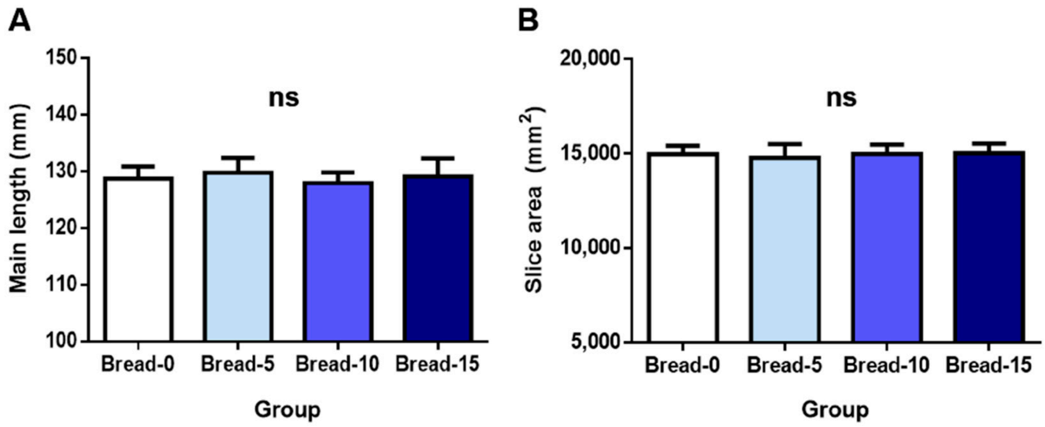

Supplementary Figure S2. Effect of FFP on bread (A) maximum height and (B) slice area.

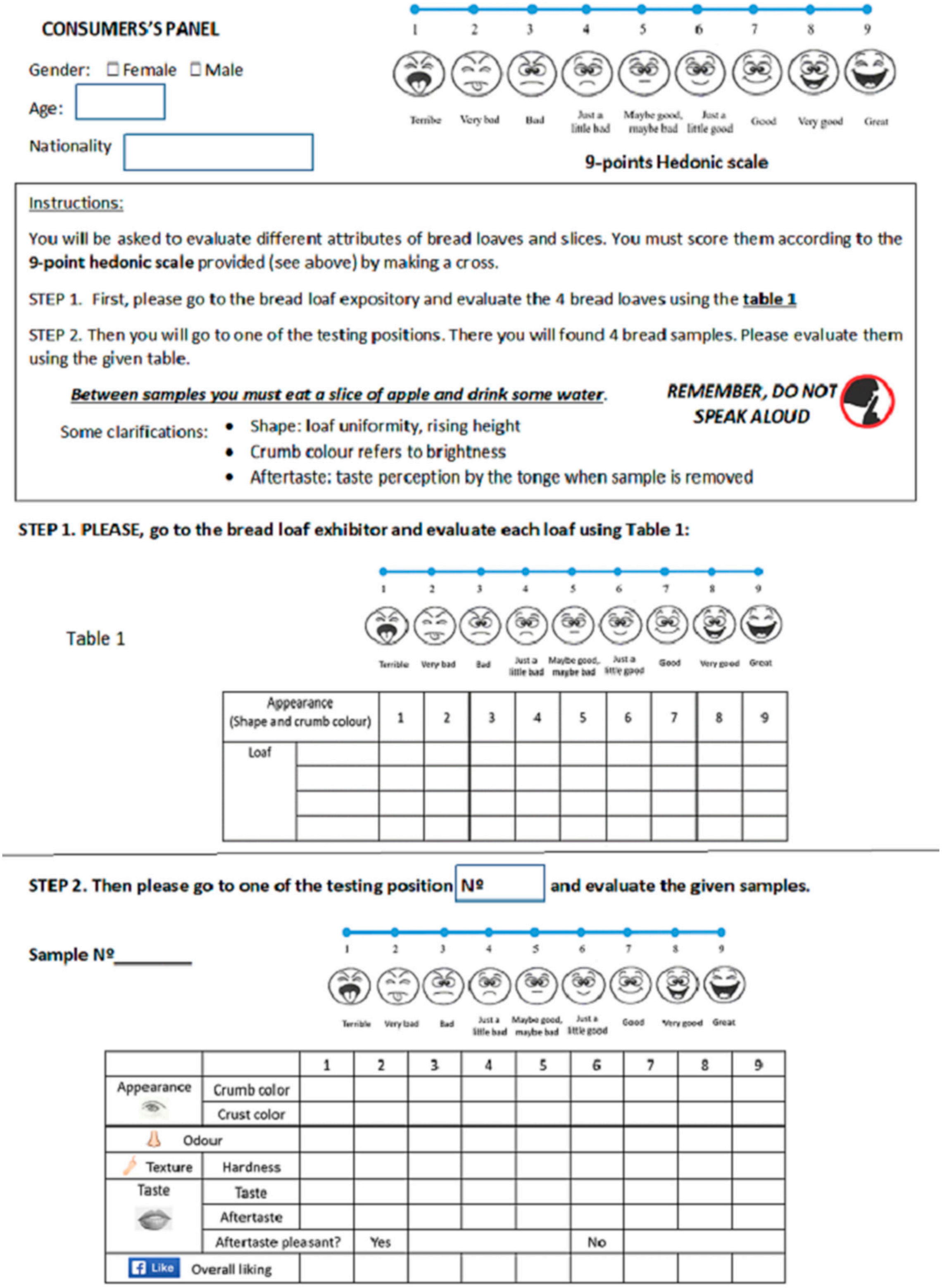

CONSUMERS'S PANEL

Gender: ☐ Female ☐ Male

Age:

Nationality

1 2 3 4 5 6 7 8 9

Terribe · Very bad · Bad · Just a little bad · Maybe good, maybe bad · Just a little good · Good · Very good · Great

9-points Hedonic scale

Instructions:

You will be asked to evaluate different attributes of bread loaves and slices. You must score them according to the **9-point hedonic scale** provided (see above) by making a cross.

STEP 1. First, please go to the bread loaf expository and evaluate the 4 bread loaves using the **table 1**

STEP 2. Then you will go to one of the testing positions. There you will found 4 bread samples. Please evaluate them using the given table.

Between samples you must eat a slice of apple and drink some water.

REMEMBER, DO NOT SPEAK ALOUD

Some clarifications:

- Shape: loaf uniformity, rising height
- Crumb colour refers to brightness
- Aftertaste: taste perception by the tonge when sample is removed

STEP 1. PLEASE, go to the bread loaf exhibitor and evaluate each loaf using Table 1:

Table 1

1 2 3 4 5 6 7 8 9

Terrible · Very bad · Bad · Just a little bad · Maybe good, maybe bad · Just a little good · Good · Very good · Great

Appearance (Shape and crumb colour)		1	2	3	4	5	6	7	8	9
Loaf										

STEP 2. Then please go to one of the testing position Nº and evaluate the given samples.

Sample Nº________

1 2 3 4 5 6 7 8 9

Terrible · Very bad · Bad · Just a little bad · Maybe good, maybe bad · Just a little good · Good · Very good · Great

		1	2	3	4	5	6	7	8	9
Appearance	Crumb color									
	Crust color									
Odour										
Texture	Hardness									
Taste	Taste									
	Aftertaste									
	Aftertaste pleasant?		Yes				No			
Like	Overall liking									

Supplementary Figure S3. Questionnaire facilitated to the panelists for the sensory evaluation of the bread samples.

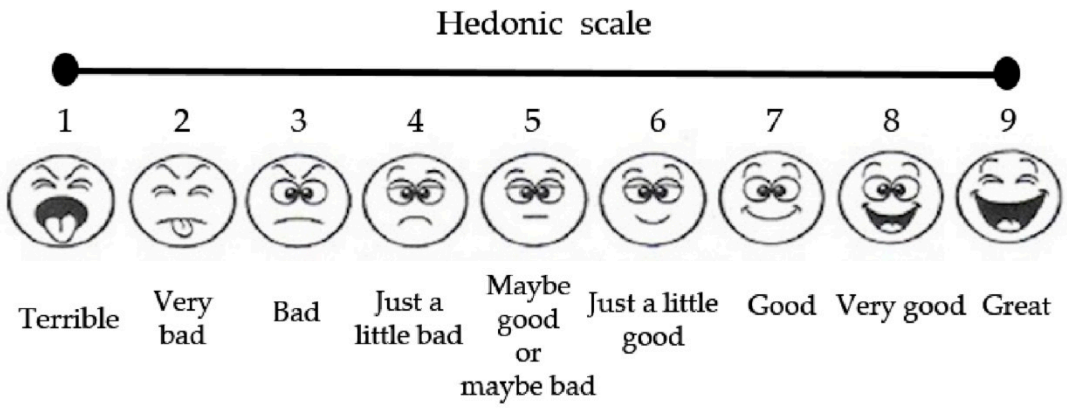

Supplementary Figure S4. Visual 9-points hedonic scale facilitated to panelists to evaluate the sensory parameters of the test samples.
